# Burden of infections and preventive strategies in patients with relapsed/refractory multiple myeloma treated with bispecific antibodies

**DOI:** 10.3389/fonc.2026.1844776

**Published:** 2026-07-20

**Authors:** Sorina-Nicoleta Badelita, Daniel-Nicolae Murariu, Andreea-Lacramioara Mohorea-Neata, Sinziana Barbu, Larisa Emilia Zidaru, Loredana Cirlan, Daniel Coriu

**Affiliations:** 1 Haematology and Bone Marrow Transplant Centre, Clinical Institute Fundeni, Bucharest, Romania; 2 Infectious Diseases Consultant for Haematology, Clinical Institute Fundeni, Bucharest, Romania; 3 Haematology Department, University of Medicine and Pharmacy Carol Davila, Bucharest, Romania

**Keywords:** anti-BCMA, anti-GPRC5D, bispecific antibodies, immunoglobulin replacement therapy, infections, multiple myeloma, vaccination

## Abstract

Bispecific antibodies(BsAbs) are a novel class of therapy for relapsed/refractory multiple myeloma (RRMM), leading to deep and durable responses in heavily pretreated patients. Their use is associated with a high burden of infections, particularly with anti-BCMA-targeting agents. This retrospective, single-center study included 50 RRMM patients treated with anti-BCMA or anti-GPRC5D BsAbs, of whom 13 received both agents because of disease progression. Standard prophylactic strategies included vaccination, antimicrobial prophylaxis, and, in selected patients, subcutaneous immunoglobulin (SCIg). Overall, 78% of patients received prior vaccinations and those with ≥3–4 vaccines exhibited the lowest infection-related mortality (23.4%, respectively 6.7%). We analyzed 121 infectious episodes recorded during the study period, of which 45.5% were severe (grade ≥3). The respiratory tract was the most affected site (65%), by viral (43%) and bacterial (45%) etiologies. Anti-BCMA BsAb were associated with a higher incidence and severity of infections, as well as a shorter median time to first infection compared with anti-GPRC5D therapy (2.3 vs 3.8 months). Infectious complications occurred throughout the course of therapy, with the highest risk observed early after treatment initiation, while remaining clinically relevant during prolonged follow-up. Infection-related mortality was the second leading cause of death (34%), after disease progression. Multivariable analyses showed that male sex and patients with high-risk disease were associated with increased infection risk.

## 1 Introduction

Immunotherapy agents, including bispecific antibodies (BsAbs), represent a new class of treatments that improve outcomes for patients with relapsed/refractory multiple myeloma (RRMM). BsAbs are approved for patients with triple-class–refractory MM who have previously received a proteasome inhibitor (PI), an immunomodulatory drug (IMiD), and an anti-CD38 monoclonal antibody (mAb). The unique dual specificity of BsAbs design enables the formation of a near-physiological immunologic synapse by engaging CD3 on T cells and surface antigens on MM cells, resulting in T-cell activation and tumour cell lysis (1).

BCMA is a transmembrane glycoprotein expressed during the late stages of B-cell differentiation and predominantly found on plasmablasts and mature plasma cells, promoting cell survival through immunoglobulin class switching and sustained antibody production. In MM, BCMA overexpression contributes to disease pathogenesis by promoting malignant plasma cell proliferation, therapeutic resistance, and immune evasion, being associated with aggressive disease features (2, 3).

Expression of the GPRC5D receptor is largely restricted to plasma cells and hard keratinized tissues, with minimal levels detected in most normal tissues. In MM, GPRC5D is frequently overexpressed in malignant plasma cells and has been associated with high-risk disease markers (4, 5).

Infections are the most frequent complication associated with novel immunotherapies and the second leading cause of death in RRMM, after disease progression itself (6). In this context, a clear understanding of the cumulative incidence and the risk factors for infections among patients with RRMM treated with BsAbs is important for guiding treatment decisions (7, 8).

Anti-BCMA agents have been associated with a higher infection rate than GPRC5D-targeting agents (9–11).Other adverse events (AEs) are class-specific related, in anti-BCMA we encounter: cytokine release syndrome (CRS) and immune effector cell-associated neurotoxicity (ICANS) - mitigated by step-up dosing administration and use of corticosteroids and tocilizumab prophylactic administration (12). GPRC5D agents have skin-related side effects such as rash, onycholysis, alopecia and dysgeusia (4).

Guidelines regarding infection prophylaxis in RRMM are scarce, and recommend antiviral prophylaxis against herpes simplex virus (HSV) and varicella zoster virus (VZV) and anti-*Pneumocystis jirovecii* prophylaxis (mainly with trimethoprim-sulfamethoxazole), some of them address the use of immunoglobulin replacement therapy (IgRT), based on the treatment administered, the grade of hypogammaglobulinemia, and the presence of recurrent infection (13) Another prophylactic method is vaccination of the patient and close contacts, which may reduce the risk of vaccine-preventable infections.

Multiple studies have shown that patients with severe hypogammaglobulinemia (IgG <400mg/dL) and severe infections that receive BsAbs could benefit from immunoglobulin replacement therapy (IgRT), which could decrease both the severity and frequency of infectious complications (9, 14–16).

IgRT in clinical practice is used as primary or secondary prophylaxis, but as of now, no clear guideline recommendations exist. Several studies have evaluated the role of IgRT as primary prophylaxis in patients receiving BsAbs therapy. However, the impact of prophylactic IgG administration on the incidence of non-severe infections (grade 1–2) remains unclear (17).

## 2 Methods

### 2.1 Study design and patient population

A retrospective observational study was conducted at a tertiary university hospital in Romania, within the Haematology Department, between October 2022 and March 2026. All adult patients with RRMM who were triple-class refractory to anti-CD38 mAb Daratumumab, IMiDs, and PIs, who received anti-BCMA or anti-GPRC5D bispecific antibodies, were included. None of the patients had received prior CAR T-cell therapy or prior exposure to bispecific antibodies before initiation of BsAb treatment. Treatment allocation was based on clinical indication and drug availability. No patients were excluded based on data completeness or follow-up duration. In case of disease progression, patients received the alternative bispecific targeting agent. A subset of patients (n=13) received both anti-BCMA and anti-GPRC5D bispecific antibodies sequentially; no separate subgroup analysis was performed for these patients.

### 2.2 Data collection

Clinical and biological data were extracted from electronic medical records, including patient demographics, disease characteristics, treatment history, prior stem cell transplantation, and BsAb received during the study period. We included vaccination history and vaccine antigens administered before treatment initiation. All infectious episodes, irrespective of severity, were recorded and graded according to the Common Terminology Criteria for Adverse Events (CTCAE, grades 1–5) (18).

Patients with suspected infection were evaluated according to our local diagnostic protocol, which included clinical assessment, complete blood count, inflammatory markers, blood cultures, and site-specific microbiological sampling. Admitted patients had weekly microbiologically screenned. In patients with respiratory symptoms, we performed the following, as clinically indicated: nasopharyngeal molecular testing for respiratory viruses and bacteria (panels, e.g., Unyvero, Quiagen), sputum testing, and targeted chest imaging. Additional diagnostic procedures, including bronchoalveolar lavage, tissue biopsy, fungal biomarkers, and histopathological examination, were performed in selected cases based on clinical presentation and clinical decision. Infectious episodes were classified as microbiologically documented when a clinically relevant pathogen was identified from blood, sterile-site specimens, or other site-directed microbiological testing, and as clinically presumed when the diagnosis was based on compatible clinical, biological and radiological findings in the absence of microbiological confirmation. Mixed infections were defined by the identification of more than one pathogen within the same infectious episode, and multisite infections by the presence of concurrent infection at more than one anatomical site. Where applicable, invasive fungal disease was classified according to EORTC/MSGERC definitions (19).

High-risk MM was defined in accordance with current recommendations from the International Myeloma Society (IMS) and the International Myeloma Working Group (IMWG) (20). Following initiation of BsAb therapy, additional data were collected on tocilizumab and corticosteroid use for CRS and ICANS.

Regarding immunoglobulin replacement therapy (IgRT), we used a subcutaneous formulation (SCIg), primarily as secondary prophylaxis in patients with high-risk disease. Other indications included the occurrence of at least three recurrent grade 1–2 infectious episodes, or at least one severe infection (grade ≥3) in the presence of severe hypogammaglobulinaemia (IgG <400 mg/dL) during the first year of the study.

All patients received antimicrobial prophylaxis against *Pneumocystis jirovecii* with trimethoprim–sulfamethoxazole and antiviral prophylaxis against HSV and VZV with acyclovir. In addition, primary prophylaxis for neutropenia was administered with filgrastim.

### 2.3 Data analysis

Data were analyzed using Python 3 (pandas, matplotlib, scipy, statsmodels, and lifelines libraries). Infection burden was modeled using Poisson regression to account for the count-based nature of infectious events. Survival analysis was performed using the Kaplan–Meier method and log-rank test. Time to first infection and infection-related mortality were analyzed using competing risk approaches with the Aalen–Johansen estimator, considering death and non-infectious death as competing events, respectively. To better characterize the temporal dynamics of infection risk, recurrent-event modeling was performed using a time-varying Cox model. Infection episodes were analyzed across predefined time intervals since treatment initiation (0–30, 30–100, 100–180, and >180 days), with the >180-day interval used as reference. In addition, the time between successive infections was analyzed to assess changes in event density over time.

Additional data exploration and visualization were performed using Microsoft Power BI.

## 3 Results

**Figure 1:**
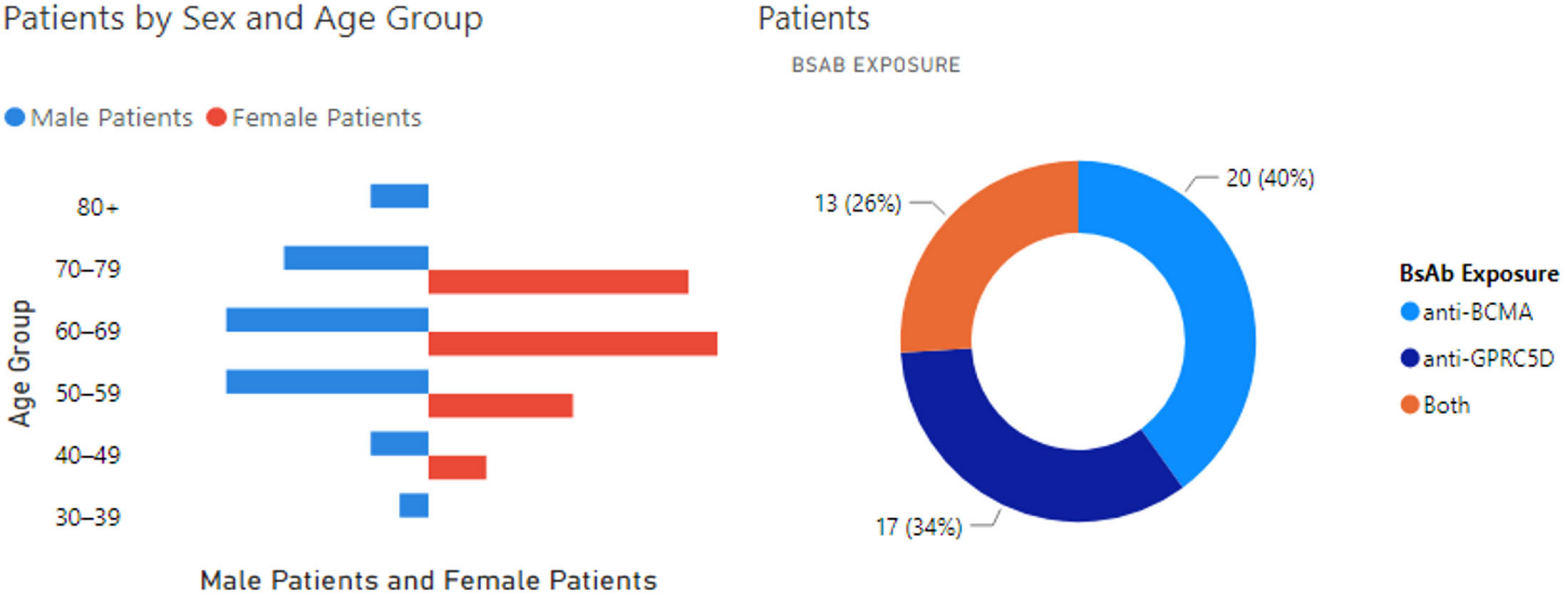
Distribution of the study population based on age/gender and BsAbs exposure.

The baseline characteristics of the study population are presented in **Table 1**. A total of 50 patients met the inclusion criteria, with a median age of 62 years. Treatment consisted of anti-BCMA therapy in 26 patients (52%) and anti-GPRC5D-targeting agents in 24 (48%), based on drug availability. Treatment switching occurred due to progressive disease (7 from anti-BCMA to anti-GPRC5D; 6 vice versa) (**Figure 1**).

**Table 1:** Baseline characteristics of the study population.

Variable	Anti-BCMA	Anti-GPRC5D	p-value
N cases	26	24	
Sex			
F	12 (46.2%)	14 (58.3%)	0.41289
M	14 (53.8%)	10 (41.7%)	0.41289
Age	56.5 (53.2-65.2)	65.5 (58.8-69.0)	0.03619*
Treatment duration	4.5 (2.0-12.1)	7.8 (2.4-14.3)	0.52787
Number of Infections	2.0 (1.0-3.0)	1.0 (0.0-2.0)	0.0869
MM Type			
IgG kappa	9 (34.6%)	7 (29.2%)	0.76664
Micromolecular kappa	3 (11.5%)	6 (25.0%)	0.2814
IgA kappa	4 (15.4%)	3 (12.5%)	1
IgG lambda	4 (15.4%)	2 (8.3%)	0.66884
Micromolecular lambda	2 (7.7%)	3 (12.5%)	0.66131
IgA lambda	2 (7.7%)	2 (8.3%)	1
IgE lambda	0 (0.0%)	1 (4.2%)	0.48
IgD lambda	1 (3.8%)	0 (0.0%)	1
IgM kappa	1 (3.8%)	0 (0.0%)	1
ASCT			
w/o	11 (42.3%)	5 (20.8%)	0.13541
1 Transplant	12 (46.2%)	17 (70.8%)	0.09342
Tandem	2 (7.7%)	2 (8.3%)	1
2 Transplants (not Tandem)	1 (3.8%)	0 (0.0%)	1
Risk			
Standard Risk	12 (46.2%)	11 (45.8%)	1
High Risk	9 (34.6%)	5 (20.8%)	0.35248
Other	5 (19.2%)	8 (33.3%)	0.33883
IgG Substitution	11 (42.3%)	8 (33.3%)	0.57005
Tocilizumab			
No	4 (15.4%)	0 (0.0%)	0.11106
Prophylactic	19 (73.1%)	23 (95.8%)	0.05045
Therapeutic	2 (7.7%)	1 (4.2%)	1
Prophylactic, Therapeutic	1 (3.8%)	0 (0.0%)	1

*For numerical variables, data are reported as median (Inter Quartile Range) and the p-values for group differences are computed by Student’s t-test. Bold values indicate statistically significant differences (p < 0.05).

All patients were triple-class refractory and had a median of 4 prior lines of therapy (range, 3-9). Regarding prior stem-cell transplant, 34 patients (68%) underwent a prior transplant, and 4 patients (8%) received tandem autologous transplantation.

Our study group included 9 high-risk patients in the anti-BCMA group and 5 patients in the anti-GRPC5D group, according to the latest IMS/IMWG consensus high-risk criteria (20). 13 patients (26%) were not included in any risk group due to a lack of cytogenetic testing.

Prophylactic tocilizumab was administered to 19 patients (73.1%) receiving anti-BCMA therapy and to 23 patients (95%) patients receiving anti-GPRC5D therapy. Grade 1 CRS occurred in 3 patients (12%) in the anti-BCMA group and one patient (6%) in the anti-GPRC5D group, while grade 1 ICANS occurred in one patient in each group.

**Figure 2:**
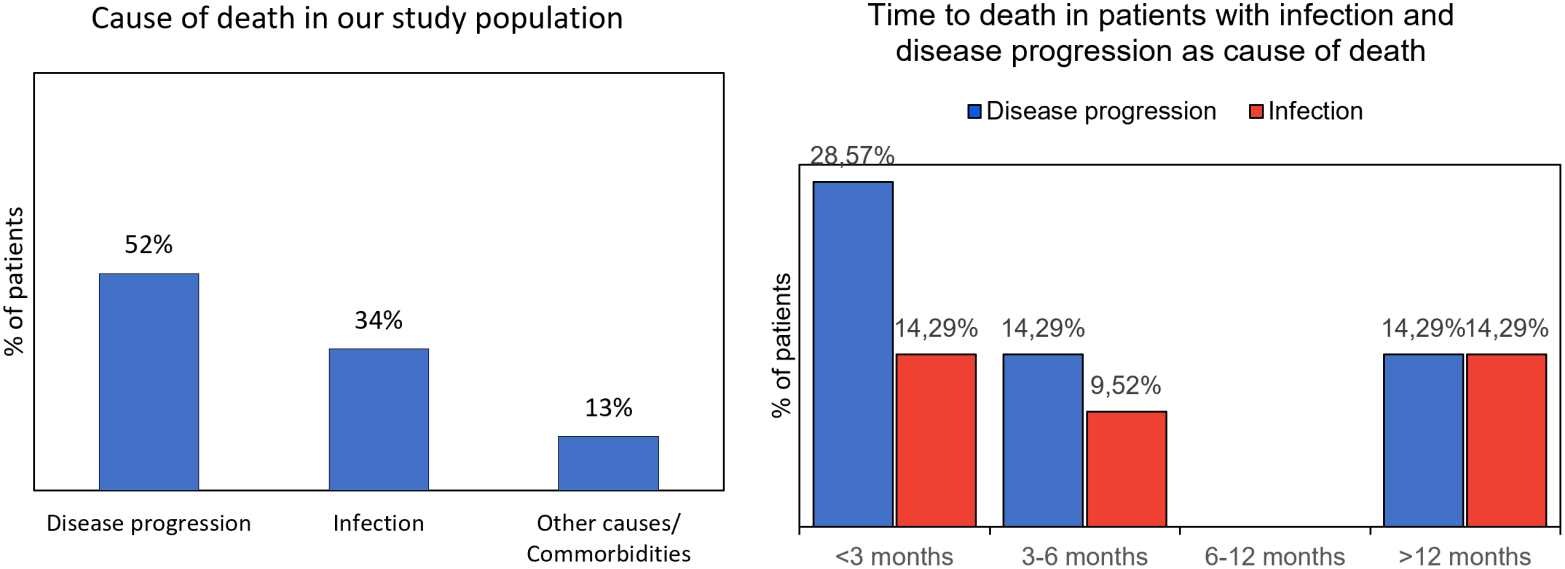
Causes of death and timing relative to SCIG therapy.

The overall mortality rate was 36.5%, with myeloma progression as the leading cause of death (52%), followed by infectious complications (34%) and other causes (13%) (**Figure 2**), which also illustrates their temporal distribution (<3, 3–6, 6–12, and >12 months).

### 3.1 Vaccines

All patients received recommendations to update their vaccines before initiation of BsAbs. In our cohort, 56% of patients had at least two doses of a SARS-CoV-2 vaccine prior to initiation of therapy. Overall, 39 patients (78%) received at least one vaccine, mostly influenza, both prior to treatment initiation and then annually, as per national recommendations. Other vaccines: 20-valent pneumococcal conjugate vaccine (with some patients having received prior immunisation with the 13-valent pneumococcal conjugate vaccine), meningococcal vaccines (serogroups ACWY and B), diphtheria–tetanus–pertussis (dTpa), and *Haemophilus influenzae* type B. Vaccination against respiratory syncytial virus (RSV), herpes zoster (inactivated formulation), and hepatitis B was less frequently administered. Hepatitis B vaccination was recommended to patients with anti-HBs titres <10 IU/mL, in the absence of prior exposure (anti-HBc negative) and with negative HBsAg. Vaccinations were administered within 1–2 weeks prior to the initiation of BsAb, although the impact of this timing on immunogenicity remains a limitation.

**Figure 3:**
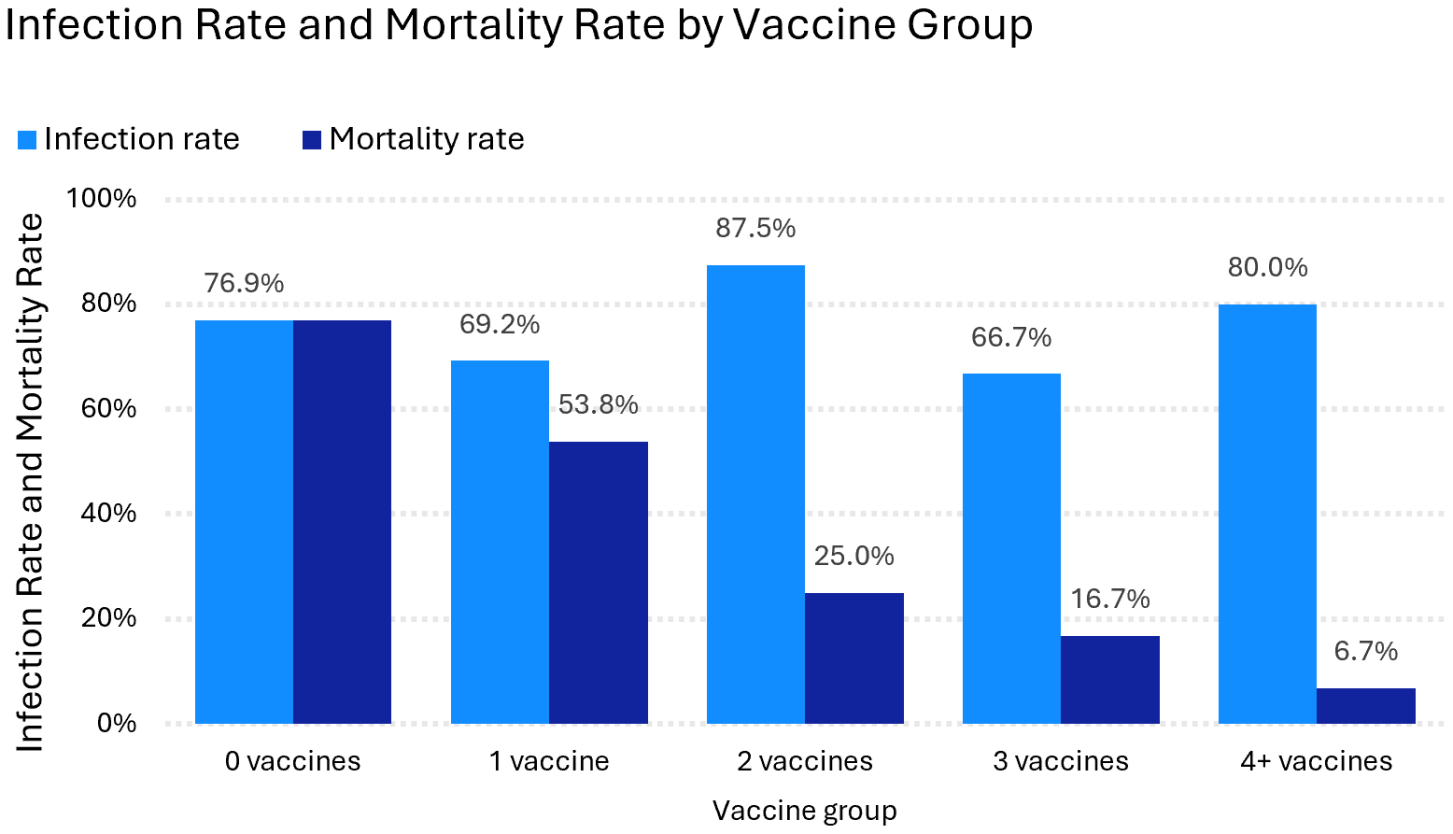
Infections and mortality rates stratified by the number of vaccines administered in patients treated with BsAbs.

We observed that patients who received ≥3–4 vaccines exhibited the lowest infection-related mortality rate, despite a persistent burden of infectious events, suggesting a potential protective effect of broader vaccination in this high-risk population (**Figure 3**).

### 3.2 Immunoglobulin replacement therapy

**Figure 4:**
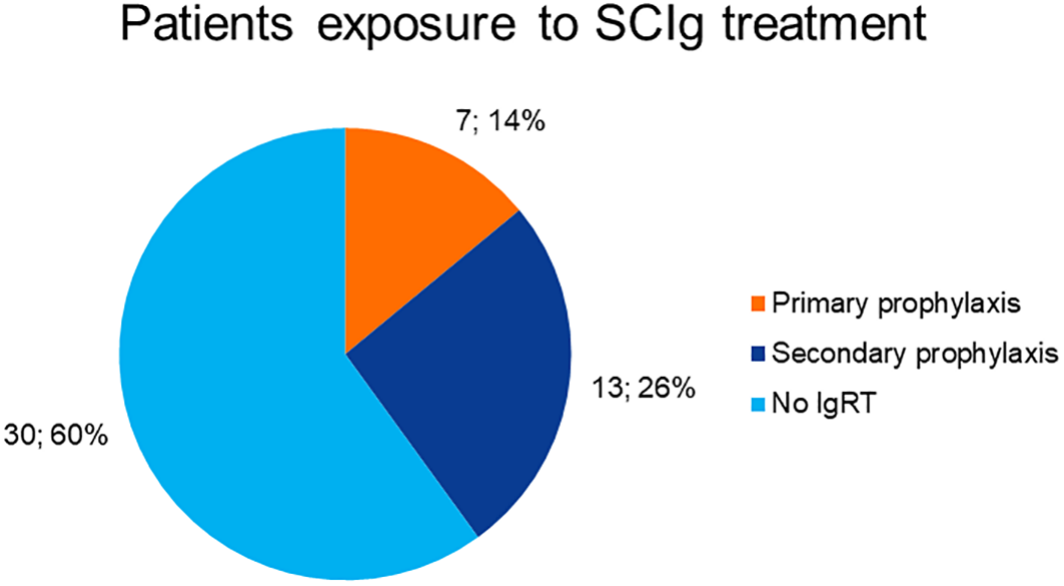
Exposure to SCIg in our study group.

Most of the patients (60%) did not receive IgRT, whereas 40% received SCIg, either as primary prophylaxis (14%) or secondary prophylaxis (26%), as shown in **Figure 4**. In our study, the use of IgRT was reserved predominantly for patients at higher risk of infectious complications or with a prior history of infection.

**Figure 5:**
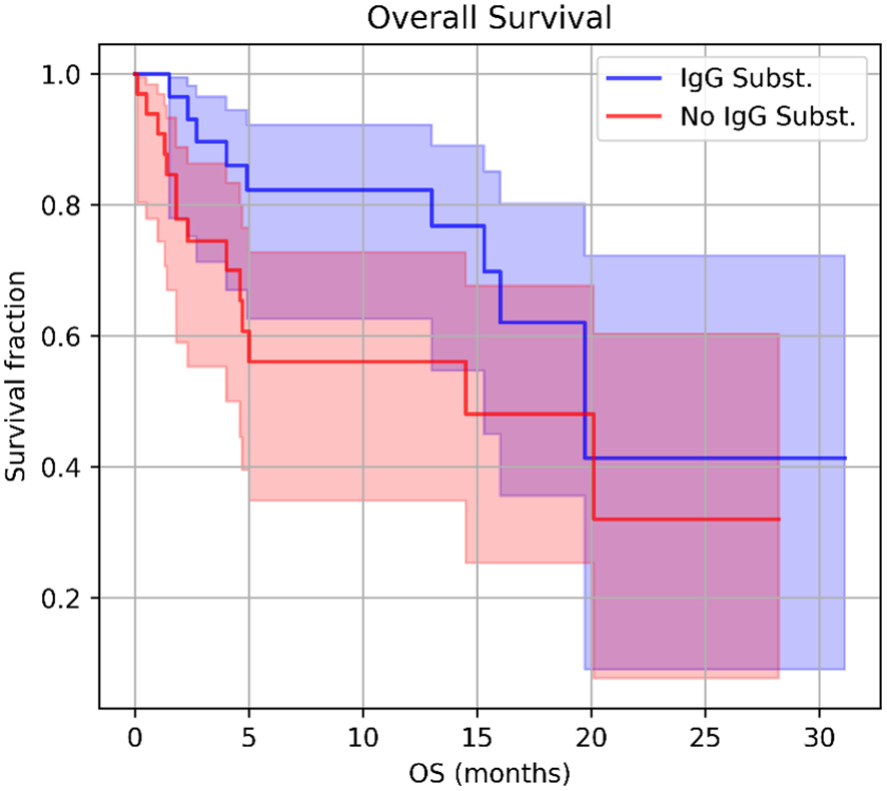
Overall survival based on SCIg substitution.

Median survival was longer in the IgRT group (19.7 months) compared with patients not receiving IgRT (14.5 months; **Figure 5**), although this difference did not reach statistical significance (p=0.0805).

**Figure 6:**
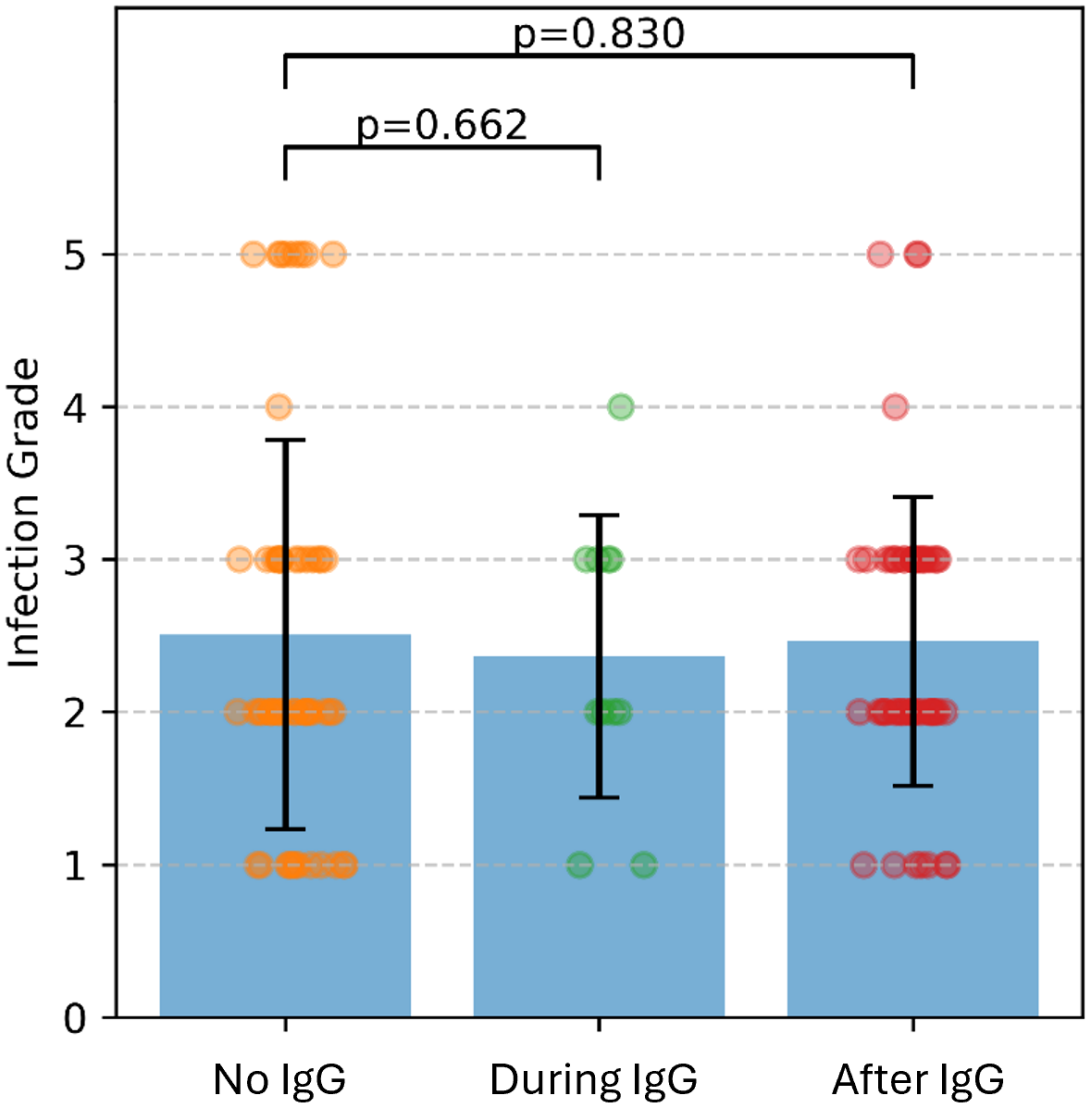
Infection incidence in patients without SCIg, during SCIg, and after SCIg therapy.

A lower incidence of infections was observed during SCIg therapy, with an apparent increase following treatment discontinuation, a pattern similar to that seen in patients not receiving IgRT. Infection severity did not differ significantly across groups (no IgRT, on IgRT, and after discontinuation), with comparable distributions of infection grades (**Figure 6**).

### 3.3 Characteristic of infections

A total of 121 infectious episodes were recorded among 49 patients included in the study. Of these, 45.5% were classified as severe (grade ≥3), while multi-pathogen and multi-site infections were relatively uncommon, occurring in 4.1% and 2.5% of episodes, respectively. Of the 121 infectious episodes, 41 (34%) were microbiologically documented and 80 (66%) were clinically presumed.

**Figure 7:**
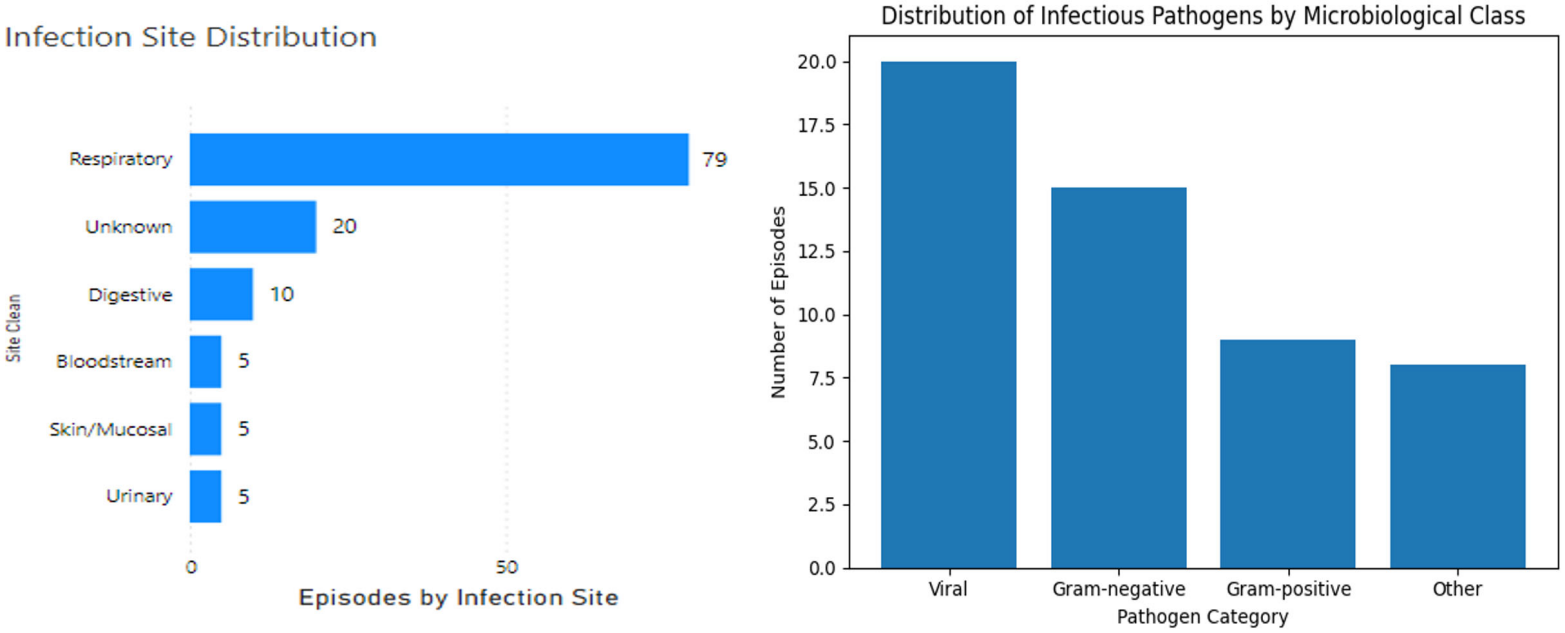
Site and pathogen distribution of infections.

Viral and bacterial pathogens accounted for the largest proportion of infections, whereas fungal infections were infrequent (**Figure 7**). The commonly identified viral agents were influenza and SARS-CoV-2. We had three cases of community-acquired measles, all classified as grade 5. Among bacterial pathogens, the most frequent Gram-negative organisms were *Escherichia coli*, *Pseudomonas* spp., and *Klebsiella* spp., while Gram-positive organisms were represented by *Streptococcus* spp. and *Staphylococcus* spp. Although rare, fungal infections are associated with severe outcomes; we report a case of a skin and soft tissue infection caused by *Fusarium* spp. that was classified as grade 5.

In terms of infection site, respiratory tract infections predominated (n=79), followed by infections of unknown origin (n=20) and gastrointestinal involvement (n=10), with bloodstream, skin and mucosal, and urinary tract infections representing a smaller proportion of cases.

Temporal analysis of infectious episodes showed variability over the study period, with higher incidence rates in late autumn and winter, particularly in November and December, whereas lower rates were recorded in summer. This trend appeared consistent across the study years.

**Figure 8:**
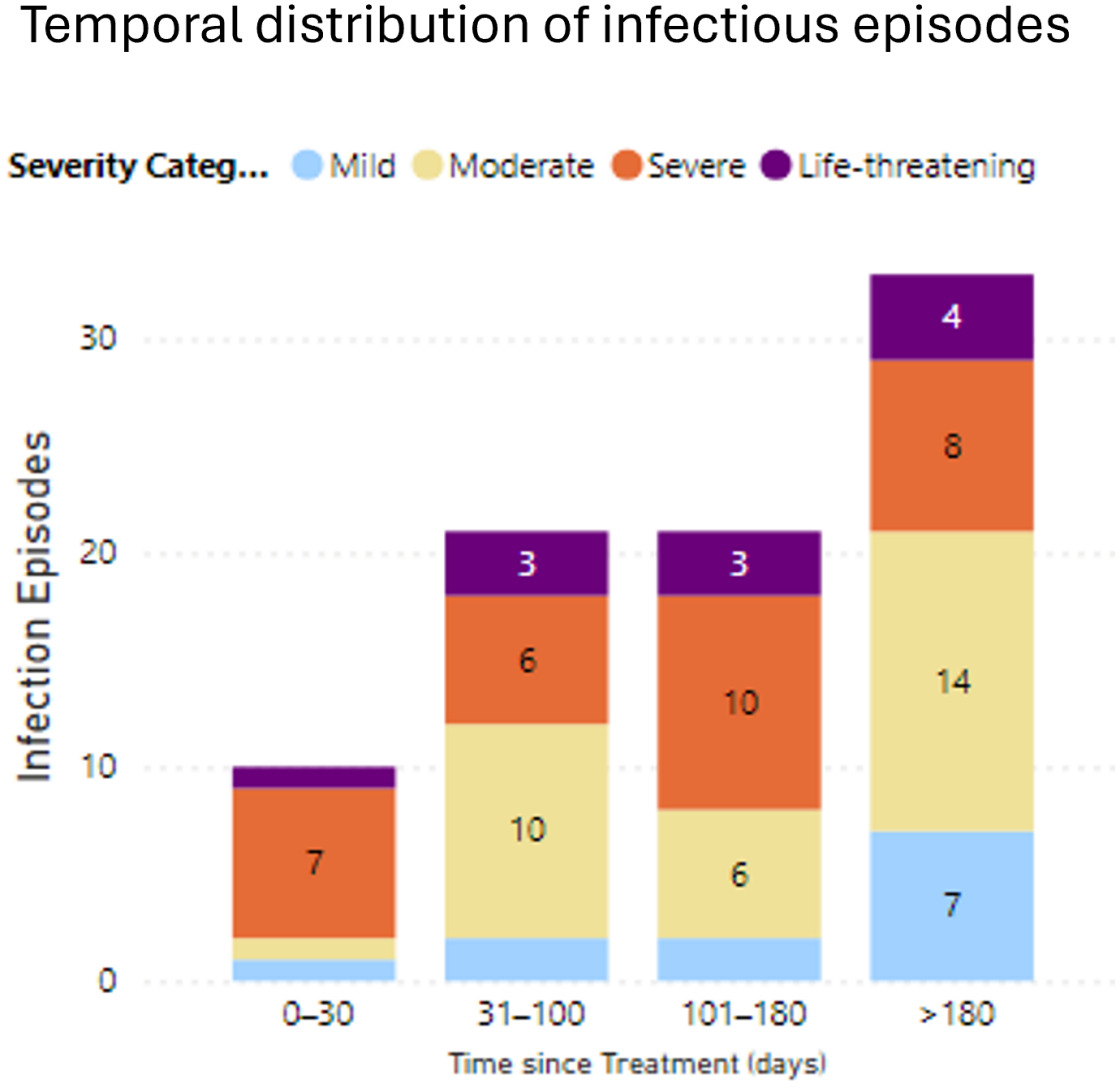
Distribution of infectious episodes by severity across treatment time intervals.

Analysis of infectious episodes during the study period highlights a substantial infectious burden following initiation of bispecific antibody therapy. Infectious events were observed across all time intervals, including beyond 180 days, with variation in severity and aetiology over time (**Figure 8**).

**Figure 9:**
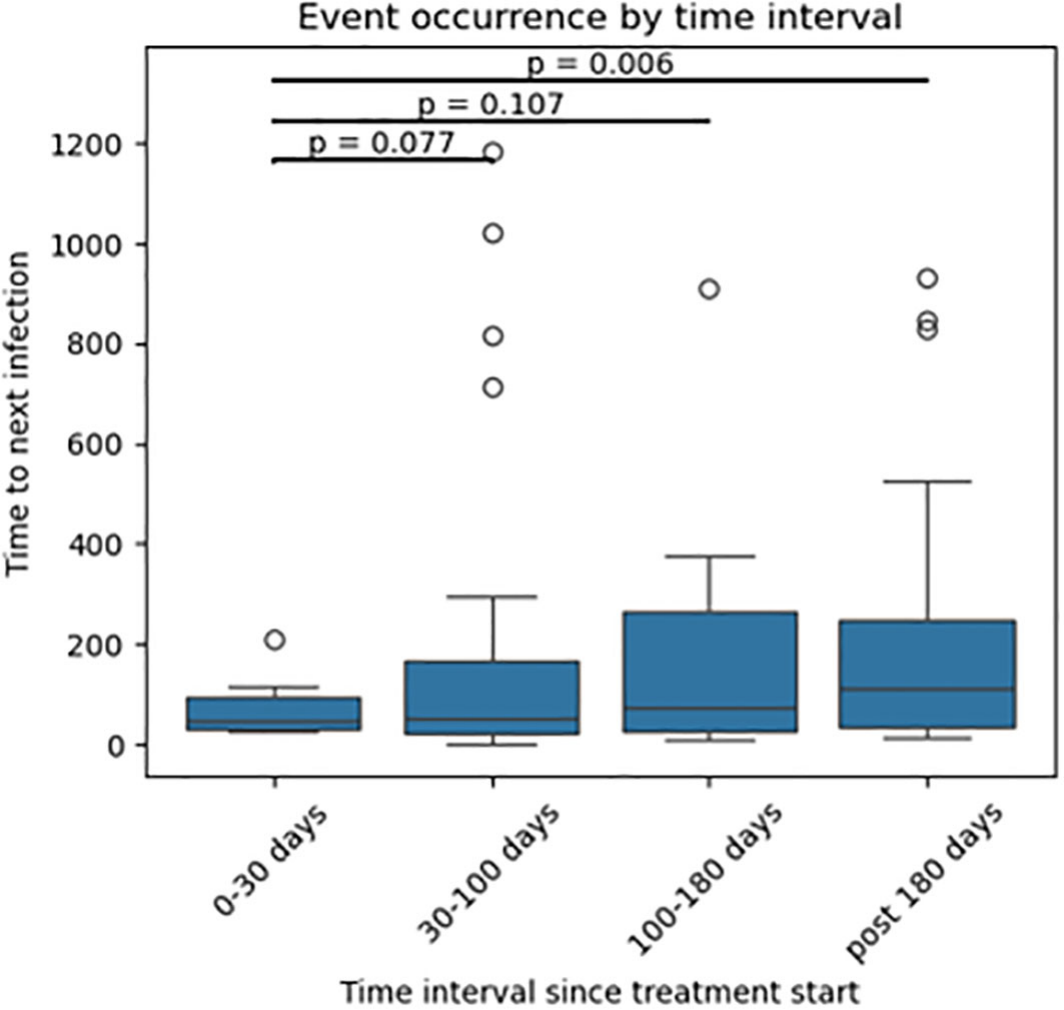
Temporal dynamics of infection risk during bispecific antibody therapy.

To further characterize temporal dynamics, recurrent-event modeling using a time-varying Cox approach demonstrated that the hazard of infection was highest during the first 30 days of treatment and declined thereafter. Consistent with this, the interval between successive infections increased over time, suggesting a lower event density despite the persistence of late infectious complications. Life-threatening infections were observed across all time intervals, including during prolonged exposure to therapy (**Figure 9**).

**Figure 10:**
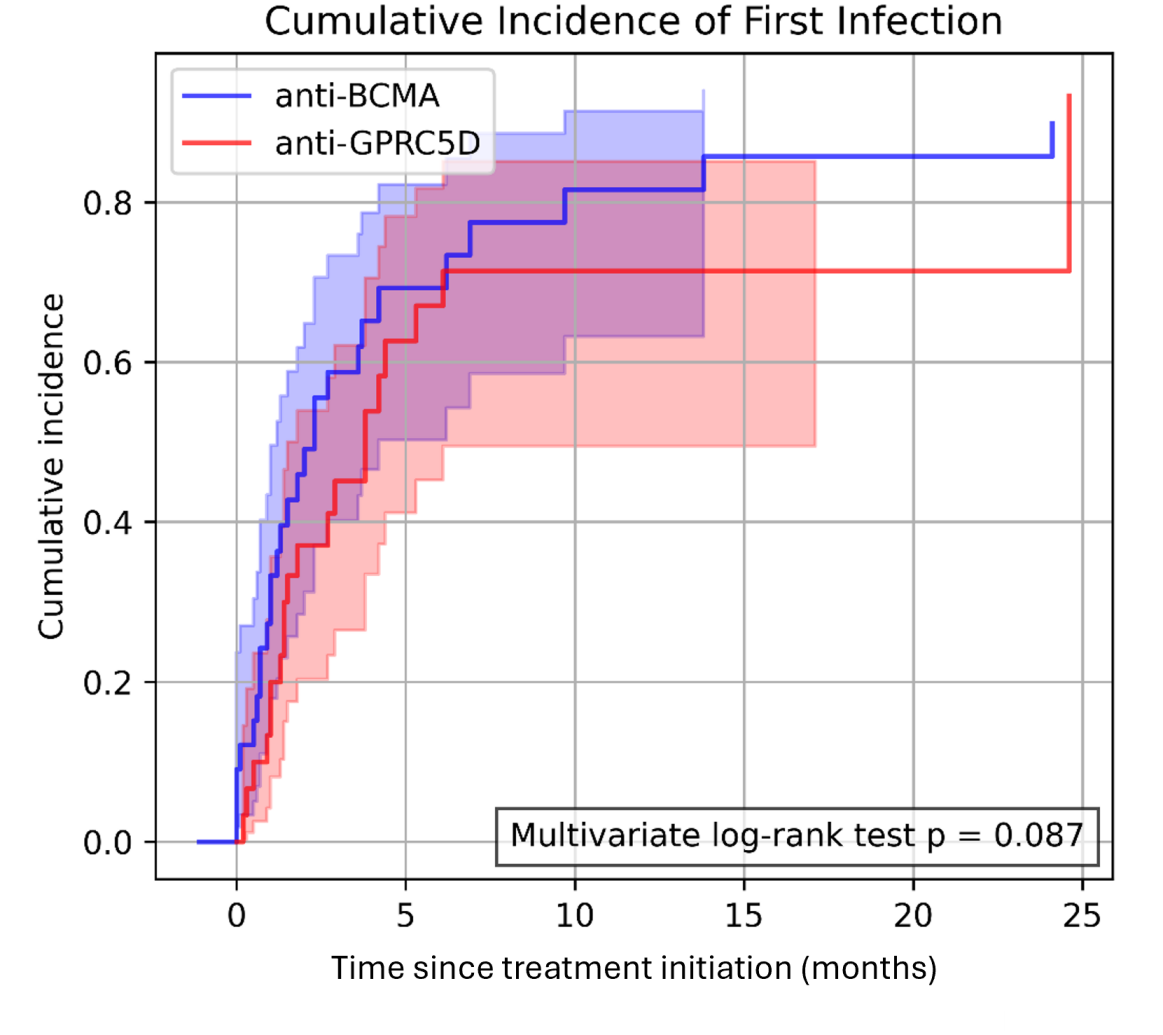
Cumulative incidence of first infection (competing risk: death).

**Figure 11:**
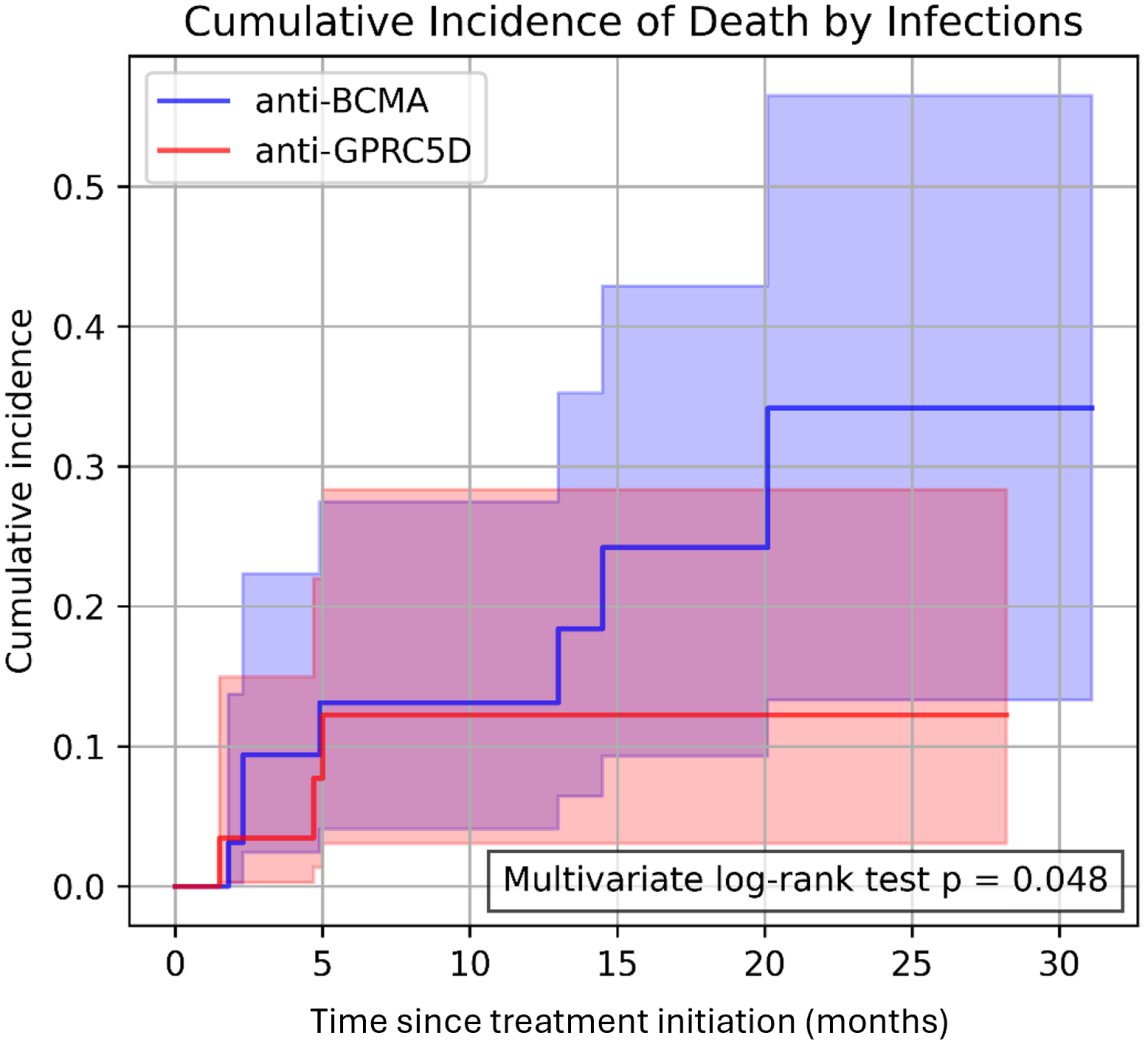
Cumulative incidence of infection-related death (competing risks: other causes).

Competing-risk analysis using cumulative incidence functions showed a numerically shorter time to first infection in patients receiving anti-BCMA compared with anti-GPRC5D therapy, with a median time to first infection of 2.3 versus 3.8 months and first-quartile times of 0.9 versus 1.4 months, respectively (**Figure 10**). For infection-related death, the cumulative incidence was higher in the anti-BCMA group; the median time to event was not reached in either group, while the first quartile was reached at 20.1 months in the anti-BCMA group and was not reached in the anti-GPRC5D group (**Figure 11**).

**Figure 12:**
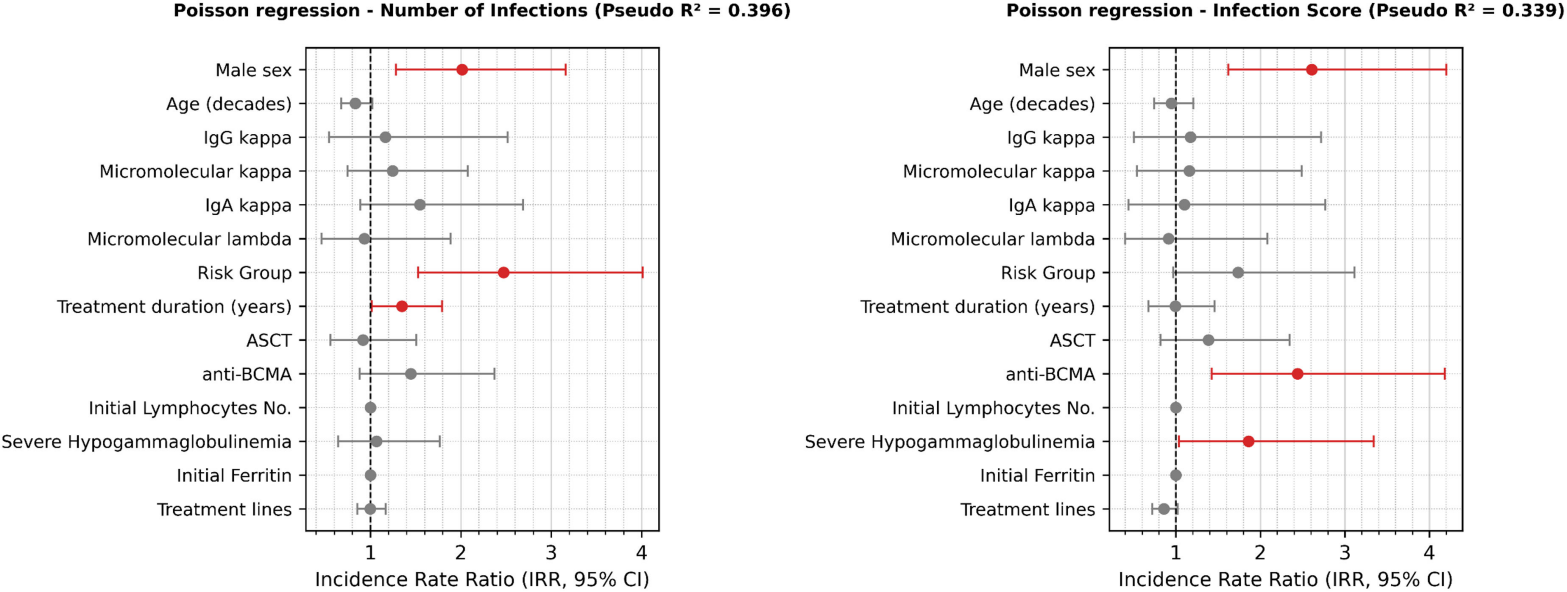
Multivariable analysis of factors associated with infection burden and severity in patients receiving BsAbs.

In multivariable Poisson regression analyses, male sex and high-risk disease status were associated with a higher incidence of infections, as reflected by both the number of infectious episodes and the cumulative infection score. Severe hypogammaglobulinemia was additionally associated with increased infection severity in the infection score model. Exposure to anti-BCMA therapy showed a trend toward increased infection burden, although effect estimates varied across models. Other clinical and biological variables, including age, baseline immunological parameters, prior autologous stem cell transplantation, and treatment-related factors, were not significantly associated with infection outcomes. Overall, the models demonstrated moderate explanatory capacity (pseudo R² = 0.396 for infection number and 0.339 for infection score), supporting the multifactorial nature of infection risk in this population (**Figure 12**).

## 4 Discussions

In our study, the incidence of all-grade infections was higher in patients receiving anti-BCMA-targeting BsAbs than in those receiving anti-GPRC5D agents, and was associated with severe episodes (p=0.0012). In our cohort, the high-risk disease group was also associated with increased infection frequency (p=0.0002) (**Figure 10**).

Furthermore, we found that the male population had a higher incidence of infectious complications (p=0.0023) and greater severity (p=0.0001). Severe hypogammaglobulinemia was also associated with a higher rate of severe infections (p= 0.0377). Baseline ferritin levels, lymphopenia, and prior transplant history didn’t have a significant impact on our study group, most likely due to the small number of patients included. Larger datasets are likely required to identify significant risk factors more accurately.

A trend towards increased infection burden was observed beyond 180 days of treatment, with a relatively stable rate of severe and life-threatening events.

The retrospective design and indication bias limit the assessment of the effect of IgRT, as therapy was administered based on clinical criteria to patients with severe or recurrent infections and/or hypogammaglobulinemia. Patients receiving SCIg were represented by those with a higher baseline infection rate (p=0.0081), and this finding should not be interpreted as evidence against IgRT efficacy, but rather as reflecting the underlying risk profiles of patients in our study group. Our results do not demonstrate a reduction in infection burden with IgRT; however, given the strong indication bias and the higher baseline risk in treated patients, these findings should not be interpreted as evidence against its efficacy. Based on real-world experience, IgRT may be considered in selected high-risk patients, particularly those with severe or recurrent infections and/or hypogammaglobulinemia, highlighting the importance of careful patient selection and timing of intervention. This approach is consistent with current international recommendations, which support the use of IgRT in patients with significant hypogammaglobulinemia and/or recurrent infections (21, 22).

Vaccination remains a key yet underutilized tool of infection prevention in immunosuppressed patients, even in RRMM treated with BsAbs. This aspect is particularly important in Romania and other Eastern European countries, where declining coverage increases vulnerability to vaccine-preventable diseases. Our National epidemiological data reported that primary pediatric vaccination uptake is approximately 68% across all included antigens, and in the general population, influenza vaccination rates remain persistently low (~6.1% in the most recent season) (23, 24). In our study, the majority of patients received at least one vaccine, and a higher number of prior vaccinations (≥3–4) was associated with the lowest observed mortality, despite a sustained burden of infections. These observations may suggest a beneficial effect of broader vaccination coverage; however, interpretation is limited by the absence of serological assessment. While vaccine-induced immune responses may be lower in this population, even partial protection is likely to translate into clinically relevant benefit. Prospective studies in patients with RRMM treated with BsAbs are needed to better define optimal vaccination, assess serological responses, and support immunization among close contacts for indirect protection, adapted to countries’ preventive strategies.

The microbiological profile observed in our cohort partially aligns with previous reports, particularly regarding the predominance of respiratory infections and the frequent involvement of viral pathogens and Gram-negative organisms in severe infections. However, another limitation of our study is the lack of antimicrobial susceptibility assessment among bacterial isolates in a setting with a high burden of antimicrobial resistance.

Another aspect was the occurrence of rare but severe events - including a fatal case of skin and soft tissue infection caused by *Fusarium* spp. and three cases of community-acquired measles, all resulting in death despite appropriate supportive care. These findings highlight the risk of opportunistic infections and vaccine-preventable pathogens, and suggest that local epidemiological factors may influence infection risk beyond what was captured in the pivotal trials.

Beyond hypogammaglobulinemia and lymphocyte depletion, infection risk in patients treated with bispecific antibodies is driven by complex immunological mechanisms involving T-cell exhaustion, cytokine dysregulation, and alterations of the bone marrow microenvironment. In our cohort, pre-emptive IL-6 blockade with tocilizumab was widely used as prophylaxis for cytokine release syndrome, contributing to a low incidence of CRS/ICANS; however, IL-6 also plays a central role in immune regulation. Chronic antigen stimulation induced by BsAbs may promote T-cell exhaustion, particularly in the context of relapsed/refractory multiple myeloma, where T-cells already exhibit impaired cytotoxicity and upregulation of inhibitory receptors. In parallel, the bone marrow niche—through stromal interactions and cytokine gradients, including IL-6 and IL-10—further promotes immunosuppression and immune escape, while supporting clonal evolution and drug resistance (25).Although IL-6 blockade may modulate this inflammatory milieu, it may also impair pathogen clearance, and its net effect on infection risk remains uncertain. Together, these mechanisms highlight that infection susceptibility in this setting extends beyond simple humoral deficiency and reflects a multifactorial disruption of immune homeostasis, underscoring the need for integrated supportive strategies and further mechanistic studies.

Although no patients were excluded, the retrospective single-center design may still have introduced selection and information biases related to real-world variability in data completeness and follow-up, beyond the standardized framework of pivotal clinical trials. In addition, the relatively small sample size limited the statistical power to detect infection risk factors and to perform robust subgroup analyses, particularly across different BsAbs and in patients receiving IgRT. Baseline heterogeneity, including differences in prior lines of therapy, disease burden, and degree of immunosuppression, may also have influenced infection risk and outcomes. This complexity is further compounded by sequential exposure to different bispecific antibodies in a subset of patients, introducing heterogeneity and potential time-dependent confounding—particularly as treatment switching was driven by disease progression and evolving infection risk—which was not specifically modeled in this analysis. Therefore, comparisons between treatment groups should be interpreted with caution.

In conclusion, BsAb therapy represents a major therapeutic advance in RRMM, leading to deep and durable responses in heavily pretreated patients. However, its use is associated with a substantial risk of infections, particularly with anti-BCMA–targeting agents. While the hazard of infection is highest early after treatment initiation, infectious complications persist throughout the course of therapy, supporting the need for extended infection monitoring beyond 6 months, as infectious complications continue to occur during long-term treatment. Preventive strategies, including vaccination, immunoglobulin replacement therapy, and antimicrobial prophylaxis, may help reduce infection-related morbidity and mortality in patients treated with BsAbs.

## Data Availability

The original contributions presented in the study are included in the article/supplementary material. Further inquiries can be directed to the corresponding authors.
